# Extracorporeal Hemoperfusion as a Potential Therapeutic Option for Severe COVID-19 patients; a Narrative Review

**Published:** 2020-08-22

**Authors:** Saeid Safari, Alireza Salimi, Alireza Zali, Alireza Jahangirifard, Ehsan Bastanhagh, Reza Aminnejad, Ali Dabbagh, Amir Hossein Lotfi, Mohammad Saeidi

**Affiliations:** 1 Functional Neurosurgery Research Center, Shohada Tajrish Neurosurgical Comprehensive Center of Excellence, Shahid Beheshti University of Medical Sciences, Tehran, Iran.; 2 Department of Anesthesiology and Critical Care, Shahid Beheshti University of Medical Sciences, Tehran, Iran.; 3 Chronic Respiratory Disease Research Center, National Research Institute of Tuberculosis and Lung Diseases (NRITLD), Shahid Beheshti University of Medical Sciences, Tehran, Iran.; 4 Department of Anesthesiology and Critical Care, Tehran University of Medical Sciences, Tehran, Iran.; 5 Department of Anesthesiology and Critical Care, Qom University of Medical Sciences, Qom, Iran.; 6 Anesthesiology Research Center, Shahid Beheshti University of Medical Sciences, Tehran, Iran.; 7 Department of Intensive Care, Laleh Hospital, Tehran, Iran.

**Keywords:** COVID-19, cytokine release syndrome, Respiratory Distress Syndrome, Adult, hemoperfusion

## Abstract

The 2019 novel coronavirus (officially known as severe acute respiratory syndrome coronavirus 2, SARS-CoV2) was first found in Wuhan, China. On February 11, 2020, the World Health Organization (WHO) has declared the outbreak of the disease caused by SARS-CoV2, named coronavirus disease 2019 (COVID-19), as an emergency of international concern. Based on the current epidemiological surveys, some COVID-19 patients with severe infection gradually develop impairment of the respiratory system, acute kidney injury (AKI), multiple organ failure, and ultimately, death. Currently, there is no established pharmacotherapy available for COVID-19. As seen in influenza, immune damage mediated by excessive production of inflammatory mediators contributes to high incidence of complications and poor prognosis. Thus, removal or blocking the overproduction of these mediators potentially aids in reducing the deleterious cytokine storm and improving critically ill patients’ outcomes. Based on previous experience of blood purification to treat cytokine storm syndrome (CSS) in severe acute respiratory syndrome (SARS) and Middle East respiratory syndrome (MERS), here we aimed to review the current literature on extracorporeal hemoperfusion as a potential therapeutic option for CSS-associated conditions, with a focus on severe COVID-19.

## Introduction

Coronavirus disease 2019 (COVID-19) is a newly recognized zoonotic respiratory infection caused by severe acute respiratory syndrome coronavirus 2 (SARS-CoV-2). It has already affected millions of people worldwide, accounting for a significant morbidity and mortality burden. Early studies, mostly coming from China, have reported that more than 20% of critically ill COVID-19 patients with pneumonia, required admission to the intensive care unit (ICU) (1). Moreover, a significant number of critical cases were reported to develop Multiple Organ Dysfunction Syndrome (MODS) and Acute Respiratory Distress Syndrome (ARDS), resulting in death within a short time (1, 2). 

A prominent modulator of sepsis, ARDS, and organ damage in critical care patients is “cytokine storm syndrome” (CSS) caused by dysregulated inflammatory response, in either the absence or presence of a pathogenic microorganism. Localized inflammation is a normal, necessary defense and repair response of body to injury or infection. This process is triggered when innate immune cells detect infection or tissue injury. Upon activation, immune cells release various pro-inflammatory cytokines including IL-1, 6, 8, 11, 12, interferon γ and TNF-α. Such pro-inflammatory response promotes production of macrophages, enhances vascular permeability, promotes coagulation, and activates extravasation of neutrophils into infected tissue. In order to restore the body homeostasis, once the pro-inflammatory stimulus is eradicated, an anti-inflammatory response (e.g. production of IL-1RA, IL-4, and IL-10) is essential to reduce the overall production of inflammatory cytokines (3). Thus, a tightly regulated self-limited protective response controls acute inflammation. However, in many life-threatening conditions such as sepsis, trauma, burn injury, severe lung injury, liver failure, pancreatitis, influenza and cytokine release syndrome, the pro/anti-inflammatory response balance is dysregulated. If homeostasis is not restored, uncontrolled pro-inflammatory response along with an unbalanced anti-inflammatory feedback causes production of excess inflammatory mediators, particularly cytokines (3).

Cytokines are a family of immunoregulatory molecules that play roles in regulation of pro and anti-inflammatory responses. Their family includes chemokines, interferons, interleukins, lymphokines, tumor necrosis factor and many others. Overproduction of these inflammatory mediators (CSS) induces severe vascular injuries, increased vascular permeability, and immense plasma leakage, leading to edema, necrosis, and cell death. These events ultimately lead to clinical symptoms including, high fever, accumulation of leukocytes and formation of blood clots in micro-vessels, hypotension, hemoconcentration, increased oxygen demand, acidosis, pulmonary edema, alveolar hemorrhage, and pleural effusion. Two common clinical sequels to CSS are ARDS and MODS.

**2. CSS in severe COVID-19**

The term “cytokine storm” in viral infection was first used in 2000, in an investigation on cytomegalovirus (4). Soon after, it began to appear more frequently in the scientific literature, showing an association with a wide variety of viral infections including Epstein-Barr virus (5), group A streptococcus (6), influenza virus (7), variola virus (8), and severe acute respiratory syndrome coronavirus (SARS-CoV) (9)(10), MERS-CoV (11), H5N1 and H1N1 influenza viruses (12, 13).

Similar to that of SARS and MERS, laboratory findings and clinical manifestations of some critical COVID-19 patients suggest a strong role for the involvement of CSS and pathophysiological sequelae (14). For instance, elevated levels of several inflammation-related biomarkers, including C-reactive protein (CRP), ferroprotein, and erythrocyte sedimentation rate (ESR) and interleukin-6 (IL-6) were reported in patients with COVID-19 pneumonia (1). Moreover, increased levels of interleukin-2 (IL-2), interleukin-7 (IL-7), interleukin-10 (IL-10) , granulocyte colony-stimulating factor (GSCF), interferon gamma-induced protein 10 (IP-10), chemokine (C-C motif) ligand 2 (CCL2), Chemokine (C-C motif) ligand 3 (CCL3), and tumor necrosis factor α (TNFα) in patients admitted to ICU compared to non-ICU patients suggests that they are related to poor prognosis (15, 16). Meanwhile, elevated levels of inflammatory indicators in the blood of COVID-19 patients are suggested to be among predictors of a fatal outcome (17).

Building on previous experience from SARS and MERS, reducing viral load by antiretroviral therapy and modulating inflammatory responses via pharmacological agents appear to be effective measures to improve the prognosis of SARS-CoV-2 infection (18-20). In organ dysfunction syndromes, when pharmacological treatment is not sufficiently effective or available, advanced methods of treatment such as mechanical ventilation and hemodynamic support are the only substitutional therapeutic strategies.

Accordingly, immunomodulatory strategies, cytokine antagonists, and mechanical removal of inflammatory mediators are already being considered or implemented in clinical practice for patients with severe COVID-19. Thus, as the COVID-19 pandemic spreads worldwide, ICU practitioners are facing a surge in critically ill patients who need acute and critical modalities.

**3. Extracorporeal blood purification (EBP) for patients with severe COVID-19**

EBP therapies are proposed as promising adjunctive treatments, designed for elimination of toxins and removal of inflammatory mediators. Even though a growing body of evidence indicates the beneficial impact of EBP use, at this stage, there are controversial reports on these techniques that should be explored (21).

Historically, EBP modalities have been recommended as complementary treatment methods for a serious overdose from a number of toxins, including salicylates, lithium, ethylene glycol, methanol, and theophylline (22-24). However, multiple extracorporeal devices have been evolved, with the intent to remove endotoxins and modulate the level of inflammatory mediators, such as cytokines/chemokines, the complement system components, and factors involved in coagulation system (21, 25-28)

The general mechanism of EBPs relies on the removal of various solutes, substances, and excessive fluid from blood through diffusion, convection, or adsorption. In these methods, patients are connected to EBP machines and their blood or plasma is pumped out of a device outside the body, passing through a column containing affinity particles. The common EBP techniques include hemodialysis, hemofiltration and hemodiafiltration, hemoperfusion, therapeutic plasma exchange, continuous renal replacement therapy, peritoneal dialysis (PD), and albumin dialysis.

Hemoperfusion therapy was first introduced in the 1940s, using an ionic resin to remove uremic toxins in dogs (29). Results from subsequent studies were sufficiently favorable to merit further research and clinical applications (30, 31). The major mechanism of hemoperfusion is elimination of circulating inflammatory mediators and endotoxins, when large volumes of the patient's blood are passed over its adsorbent substance. Circulating endotoxin or inflammatory mediators are attached to the highly adsorptive membrane through hydrophobic, ionic, and van der Waals interaction; thus, being eliminated from circulation (25-27). The sorbent system is made up of a biocompatible fixed bed, or cartridge, which contains the adsorbent particles. There are two major types of adsorbent materials, including activated charcoal and resins (e.g. hydrocarbon polymer, polystyrene). Charcoal has greater affinity for water-soluble molecules, whereas resins have higher affinity toward lipid-soluble molecules (e.g., glutethimide and methaqualone). Particular characteristics of solutes such as molecular size, chemical affinity, and their distribution volume in the body determine the efficacy of the hemoperfusion.

Over the years, improvement of biocompatibility and adsorption capacity of hemoperfusion systems increased their use in the critical care settings. Therefore, even though, classical application of hemoperfusion was elimination of drug or chemical toxics from circulation, recently treatment of inflammatory conditions has been suggested as a potential use for hemoperfusion. Regardless of limited evidence, hemoperfusion administration for removal of inflammatory mediators from the bloodstream has been reported to have a beneficial impact on the treatment of dysregulated inflammatory conditions (32). For instance, application of this technique has been reported to have favorable effects in several cases of influenza (especially H1N1 and H5N1 subtypes) (33, 34). Moreover, hemoperfusion has successfully been used to immediately ameliorate severe CSS and prevent MOD, pneumonia, and hydrosarca caused by chimeric antigen receptor (CAR) T cell therapy (35, 36). 

Though not definitive, the profile of cytokine and inflammation in SARS-CoV 2 infection suggests that a severe dysregulated inflammatory response is a fundamental problem in quite some critically ill COVID-19 patients (37-40). Therefore, the rationale of using extracorporeal organ support therapies including hemoperfusion, with efficient sorbent cartridges for removal of cytokines and other inflammatory circulating mediators, should be considered in critically-ill COVID-19 patients (41). To date, various centers in different countries including Italy, China, USA, Germany, and Iran have reported or are investigating the beneficial effects of different hemoperfusion systems, including HA380/HA330 cartridges, CytoSorb, and polymyxin B immobilized fiber column in treatment of critically-ill COVID-19 patients. 

The HA type hemoperfusion cartridges (HA130, HA230, HA330 and HA380) (Jafron, China) are among the widely used HA devices in China. The cartridges contain highly biocompatible sorbents and neutro-macroporous resin made of styrene-divinylbenzene copolymer. HA 330 and HA 380 cartridges are mainly used in acute inflammatory conditions. Their adsorbing beads’ pore size ranged from 500 D to 60 kD, giving them the ability to absorb various medium-sized factors, including most inflammatory cytokines (IL-1, IL-6, IL-8, and TNF-a) (42). The results of multiple studies have demonstrated that application of HA 330 to eliminate circulating and alveolar levels of pro-inflammatory cytokines in severe sepsis, septic shock, or acute lung injury patients significantly improved patients’ hemodynamics, reduced the length of intensive care unit stay, and intensive care unit mortality (42-44). Currently, a clinical trial is ongoing (IRCT20200317046797N5, Imam Reza Hospital, Tabriz, Iran) for evaluating the effectiveness of HA 330 to remove cytokines in patients admitted with severe forms of COVID-19 and before intubation. Severity of pneumonia based on CT scan, ARDS, mortality rate, and hospitalization duration are the main variables that will be evaluated in this study. 

Meanwhile, a number of studies, mostly published in Europe, have evaluated the clinical use of other hemoperfusion type, CytoSorb cartridges (CytoSorbents Corporation, NJ, USA), in the management of conditions associated with elevated inflammatory mediators (45). CytoSorb cartridges contain biocompatible highly porous copolymers, capable of binding a broad spectrum of hydrophobic compounds with a molecular weight between 10 and 55 kDa. Even though most cytokines and other inflammatory mediators reside within this molecular weight range, their removal is concentration dependent. Thus, low cytokine plasma concentrations are not removed efficiently, but high cytokine plasma levels are reduced effectively (46, 47). While blood is passed through the absorbent bed, proteins and other hydrophobic molecules less than approximately 60 kDa enter the device pores and attach onto the surface of the hydrophobic polymer via non-polar interactions, hydrogen bonding, and van der Waals forces. To date, a large number of experimental and clinical data, mostly from case reports and case series, have introduced CytoSorb as an effective rescue therapy for removal of inflammatory cytokines and achievement of hemodynamic stabilization in critically ill patients with septic shock and kidney failure (47-49). The positive results from these studies have led to the consideration of CytoSorb application in critically ill COVID-19 patients. In Italy, the formal recommendation is made by the Italy Brescia Renal COVID Task Force and a publication by the Italian Society of Nephrology and ERA-EDTA, to specifically use CytoSorb in severe COVID-19 patients with Stage 3 renal failure on continuous renal replacement therapy (CRRT) (50). Meanwhile, the emergency use of CytoSorb for ICU patients with confirmed or imminent respiratory failure has been approved by US Food and Drug Administration (FDA), issuing an Emergency Use Authorization (EUA) (51, 52). Also, the recent National Guidelines on adult COVID-19 patients from Panama recommend CytoSorb therapy if patients have either refractory shock, or have severe or refractory respiratory failure requiring high ventilator support or extracorporeal membrane oxygenation (53). The recent Handbook of COVID-19 Prevention and Treatment from Zhejiang University School of Medicine, China, is also recommending blood purification to treat cytokine storm in critical cases of COVID-19 infection (54). 

In addition to several recommendations for CytoSorb therapy in COVID-19 patients, Health Canada’s Interim Order has recently approved the use of the Spectral’s Toraymyxin™ (PMX) hemoperfusion cartridge to treat COVID-19 (55), particularly in cases with ARDS, diffuse alveolar damage or difficulty maintaining oxygenation, in the presence of hypotensive shock. Toraymyxin is composed of covalently immobilized polymyxin B (PMX-B) fiber as an absorbent bed. PMX-B is an antibiotic, which is known to bind to endotoxin, selectively. Even though the efficacy of PMX-B immobilized fiber columns for direct hemoperfusion in CSS related conditions is still debated (56-58), they have already shown promising results in treatment of avian flu (H5N1) and swine flu (H1N1), which cause seasonal epidemic and occasional pandemic outbreaks. Application of PMX-B to remove endotoxins from influenza patients showed a significant improvement in chest x-ray results and lung function; in addition, it led the patients to earlier weaning from ventilators (59). 

According to Spectral Medical Inc, a therapeutic company focused on the development of a treatment for septic shock, elevated levels of endotoxin activity, as measured by their FDA-approved Endotoxin Activity Assay (EAA), have been identified in COVID-19 patients in Japan, Italy, and the US (60). Since endotoxin is the primary driver of the CSS, its elimination by the PMX-B cartridge is supposed to reduce circulating levels of cytokines. Thus, hemoperfusion with PMX-B is expected to be an effective method to reduce inflammatory mediators by elimination of endotoxins. An ongoing clinical trial (NCT04352985) is currently investigating the efficacy of Toraymyxin PMX cartridge with a focus on safety of its application for patients with septic shock and COVID-19.

To sum up, hemoperfusion for solute removal has been used for years. Even though, early indication for hemoperfusion was severe intoxication, in recent years these devices have evolved for the purpose of immunomodulation in acute conditions like sepsis. With a multitude of studies and researches indicating that patients with COVID-19 experience an immune response dysregulation and CSS, this blood purification technique is likely to be a feasible treatment modality in the case of severe SARS-CoV2 infection. However, the real impact of hemoperfusion on the patient's clinical course has yet to be determined. Thus, in future studies, patient selection should be approached with caution and consideration.

## References

[1] Chen N, Zhou M, Dong X, Qu J, Gong F, Han Y (2020). Epidemiological and clinical characteristics of 99 cases of 2019 novel coronavirus pneumonia in Wuhan, China: a descriptive study. Lancet (London, England).

[2] Yang X, Yu Y, Xu J, Shu H, Xia J, Liu H (2020). Clinical course and outcomes of critically ill patients with SARS-CoV-2 pneumonia in Wuhan, China: a single-centered, retrospective, observational study. The Lancet Respiratory medicine.

[3] Anand D, Ray S, Bhargava S, Das S, Garg A, Taneja S (2014). Proinflammatory versus anti-inflammatory response in sepsis patients: looking at the cytokines. Critical Care.

[4] Barry S, Johnson M, Janossy G (2000). Cytopathology or immunopathology? The puzzle of cytomegalovirus pneumonitis revisited. Bone marrow transplantation.

[5] Imashuku S (2002). Clinical features and treatment strategies of Epstein–Barr virus-associated hemophagocytic lymphohistiocytosis. Critical reviews in oncology/hematology.

[6] Bisno A, Brito M, Collins C (2003). Molecular basis of group A streptococcal virulence. The Lancet infectious diseases.

[7] Yokota S (2003). Influenza-associated encephalopathy--pathophysiology and disease mechanisms. Nihon rinsho Japanese journal of clinical medicine.

[8] Jahrling PB, Hensley LE, Martinez MJ, LeDuc JW, Rubins KH, Relman DA (2004). Exploring the potential of variola virus infection of cynomolgus macaques as a model for human smallpox. Proceedings of the National Academy of Sciences.

[9] Huang KJ, Su IJ, Theron M, Wu YC, Lai SK, Liu CC (2005). An interferon‐γ‐related cytokine storm in SARS patients. Journal of medical virology.

[10] Pedersen SF, Ho Y-C (2020). SARS-CoV-2: a storm is raging. The Journal of clinical investigation.

[11] Lau SK, Lau CC, Chan K-H, Li CP, Chen H, Jin D-Y (2013). Delayed induction of proinflammatory cytokines and suppression of innate antiviral response by the novel Middle East respiratory syndrome coronavirus: implications for pathogenesis and treatment. Journal of General Virology.

[12] Yuen K, Wong S (2005). Human infection by avian influenza A H5N1. Hong Kong Medical Journal.

[13] Österlund P, Pirhonen J, Ikonen N, Rönkkö E, Strengell M, Mäkelä SM (2010). Pandemic H1N1 2009 Influenza A Virus Induces Weak Cytokine Responses in Human Macrophages and Dendritic Cells and Is Highly Sensitive to the Antiviral Actions of Interferons. Journal of Virology.

[14] Pollard HB, Pollard BS, Pollard JR Classical drug digitoxin inhibits influenza cytokine storm, with implications for COVID-19 therapy. bioRxiv.

[15] Gong J, Dong H, Xia SQ, Huang YZ, Wang D, Zhao Y (2020). Correlation analysis between disease severity and inflammation-related parameters in patients with COVID-19 pneumonia. MedRxiv.

[16] Huang C, Wang Y, Li X, Ren L, Zhao J, Hu Y (2020). Clinical features of patients infected with 2019 novel coronavirus in Wuhan, China. The lancet.

[17] Ruan Q, Yang K, Wang W, Jiang L, Song J (2020). Clinical predictors of mortality due to COVID-19 based on an analysis of data of 150 patients from Wuhan, China. Intensive care medicine.

[18] Stockman LJ, Bellamy R, Garner P (2006). SARS: systematic review of treatment effects. PLoS medicine.

[19] Falzarano D, De Wit E, Rasmussen AL, Feldmann F, Okumura A, Scott DP (2013). Treatment with interferon-α2b and ribavirin improves outcome in MERS-CoV–infected rhesus macaques. Nature medicine.

[20] Omrani AS, Saad MM, Baig K, Bahloul A, Abdul-Matin M, Alaidaroos AY (2014). Ribavirin and interferon alfa-2a for severe Middle East respiratory syndrome coronavirus infection: a retrospective cohort study. The Lancet Infectious Diseases.

[21] Ankawi G, Neri M, Zhang J, Breglia A, Ricci Z, Ronco C (2018). Extracorporeal techniques for the treatment of critically ill patients with sepsis beyond conventional blood purification therapy: the promises and the pitfalls. Critical Care.

[22] Goldfarb D, Matalon D Principles and techniques applied to enhance elimination Goldfrank's toxicologic emergencies.

[23] Locket S (1970). Haemodialysis in the treatment of acute poisoning. Proceedings of the Royal Society of Medicine.

[24] Doolan PD, Walsh WP, Wishinsky H (1951). Acetylsalicylic acid intoxication; a proposed method of treatment. Journal of the American Medical Association.

[25] Fiore B, Soncini M, Vesentini S, Penati A, Visconti G, Redaelli A (2006). Multi-scale analysis of the toraymyxin adsorption cartridge Part II: computational fluid-dynamic study. The International journal of artificial organs.

[26] Vesentini S, Soncini M, Zaupa A, Silvestri V, Fiore GB, Redaelli A (2006). Multi-scale analysis of the toraymyxin adsorption cartridge Part I: molecular interaction of polymyxin B with endotoxins. The International journal of artificial organs.

[27] Ronco C, Piccinni P, Kellum J (2010). Rationale of extracorporeal removal of endotoxin in sepsis: theory, timing and technique. Contributions to nephrology.

[28] Navas A, Ferrer R, Martinez ML, Goma G, Gili G, Masip J (2018). Impact of hemoperfusion with polymyxin B added to hemofiltration in patients with endotoxic shock: a case-control study. Annals of intensive care.

[29] Muirhead EE, Reid AF (1948). A resin artificial kidney. The Journal of laboratory and clinical medicine.

[30] Pallotta AJ, Koppanyi T (1960). The use of ion exchange resins in the treatment of phenobarbital intoxication in dogs. The Journal of pharmacology and experimental therapeutics.

[31] SCHREINER GE (1958). The Role of Hemodialysis (Artificial Kidney) in Acute Poisoning. AMA Archives of Internal Medicine.

[32] Bonavia A, Groff A, Karamchandani K, Singbartl K (2018). Clinical Utility of Extracorporeal Cytokine Hemoadsorption Therapy: A Literature Review. Blood purification.

[33] Takeda S, Munakata R, Abe S, Mii S, Suzuki M, Kashiwada T (2010). Hypercytokinemia with 2009 pandemic H1N1 (pH1N1) influenza successfully treated with polymyxin B-immobilized fiber column hemoperfusion. Intensive care medicine.

[34] Binh NG, Manabe T, Co DX, Tuan ND, Thach PT, Kudo K (2015). Polymyxin-B-immobilized-fiber column hemoperfusion with oseltamivir treatment for ARDS due to influenza H1N1/09. Respirology case reports.

[35] Liu Y, Chen X, Wang D, Li H, Huang J, Zhang Z (2018). Hemofiltration Successfully Eliminates Severe Cytokine Release Syndrome Following CD19 CAR-T-Cell Therapy. Journal of immunotherapy (Hagerstown, Md : 1997).

[36] Stahl K, Schmidt BMW, Hoeper MM, Skripuletz T, Möhn N, Beutel G (2020). Extracorporeal cytokine removal in severe CAR-T cell associated cytokine release syndrome. Journal of Critical Care.

[37] Tay MZ, Poh CM, Rénia L, MacAry PA, Ng LFP (2020). The trinity of COVID-19: immunity, inflammation and intervention. Nature Reviews Immunology.

[38] Mehta P, McAuley DF, Brown M, Sanchez E, Tattersall RS, Manson JJ (2020). COVID-19: consider cytokine storm syndromes and immunosuppression. Lancet (London, England).

[39] Birndt S, Schenk T, Heinevetter B, Brunkhorst FM, Maschmeyer G, Rothmann F (2020). Hemophagocytic lymphohistiocytosis in adults: collaborative analysis of 137 cases of a nationwide German registry. Journal of cancer research and clinical oncology.

[40] Hormati A, Ghadir MR, Zamani F, Khodadadi J, Khodadust F, Afifian M (2020). Are there any association between COVID-19 severity and immunosuppressive therapy?. Immunol Lett.

[41] Ronco C, Navalesi P, Vincent J (2020). Coronavirus epidemic: Preparing for extracorporeal organ support in intensive care. Lancet Respir Med.

[42] Ankawi G, Fan W, Pomarè Montin D, Lorenzin A, Neri M, Caprara C (2019). A New Series of Sorbent Devices for Multiple Clinical Purposes: Current Evidence and Future Directions. Blood purification.

[43] Huang Z, Wang SR, Su W, Liu JY (2010). Removal of humoral mediators and the effect on the survival of septic patients by hemoperfusion with neutral microporous resin column. Therapeutic apheresis and dialysis : official peer-reviewed journal of the International Society for Apheresis, the Japanese Society for Apheresis, the Japanese Society for Dialysis Therapy.

[44] Huang Z, Wang SR, Yang ZL, Liu JY (2013). Effect on extrapulmonary sepsis-induced acute lung injury by hemoperfusion with neutral microporous resin column. Therapeutic apheresis and dialysis : official peer-reviewed journal of the International Society for Apheresis, the Japanese Society for Apheresis, the Japanese Society for Dialysis Therapy.

[45] Poli EC, Rimmelé T, Schneider AG (2019). Hemoadsorption with CytoSorb(®). Intensive care medicine.

[46] Basu R, Pathak S, Goyal J, Chaudhry R, Goel RB, Barwal A (2014). Use of a novel hemoadsorption device for cytokine removal as adjuvant therapy in a patient with septic shock with multi-organ dysfunction: A case study. Indian journal of critical care medicine : peer-reviewed, official publication of Indian Society of Critical Care Medicine.

[47] Kogelmann K, Jarczak D, Scheller M, Drüner M (2017). Hemoadsorption by CytoSorb in septic patients: a case series. Critical Care.

[48] Hinz B, Jauch O, Noky T, Friesecke S, Abel P, Kaiser R (2015). CytoSorb, a novel therapeutic approach for patients with septic shock: a case report. The International journal of artificial organs.

[49] Calabrò MG, Febres D, Recca G, Lembo R, Fominskiy E, Scandroglio AM (2019). Blood Purification With CytoSorb in Critically Ill Patients: Single-Center Preliminary Experience. Artificial organs.

[50] Nephrology ISo, Alberici F GESTIONE DEL PAZIENTE IN DIALISI E CON TRAPIANTO DI RENE IN CORSO DI INFEZIONE DA CORONAVIRUS COVID-19 2020.

[51] FDA has authorized the emergency use of CytoSorb 300 mL device: CytoSorb 300mL device is manufactured under and ISO 13485 and CE Mark approved 2020.

[52] Friesecke S, Trager K, Schittek GA, Molnar Z, Bach F, Kogelmann K (2019). International registry on the use of the CytoSorb(R) adsorber in ICU patients : Study protocol and preliminary results. Medizinische Klinik, Intensivmedizin und Notfallmedizin.

[53] Intensiva APdMCyT GUÍAS NACIONALES DE ATENCIÓN DE PACIENTES ADULTOS COVID-19 VERSION 2.0 2019.

[54] Commission CNH (2020). Chinese Clinical Guidance for COVID-19 Pneumonia Diagnosis and Treatment (7th Edition).

[55] Canada Go List of medical devices for expanded use in relation to the COVID-19 pandemic 2020.

[56] Dellinger RP, Bagshaw SM, Antonelli M, Foster DM, Klein DJ, Marshall JC (2018). Effect of Targeted Polymyxin B Hemoperfusion on 28-Day Mortality in Patients With Septic Shock and Elevated Endotoxin Level: The EUPHRATES Randomized Clinical Trial. Jama.

[57] Klein DJ, Foster D, Walker PM, Bagshaw SM, Mekonnen H, Antonelli M (2018). Polymyxin B hemoperfusion in endotoxemic septic shock patients without extreme endotoxemia: a post hoc analysis of the EUPHRATES trial. Intensive care medicine.

[58] Nakamura Y, Kitamura T, Kiyomi F, Hayakawa M, Hoshino K, Kawano Y (2017). Potential survival benefit of polymyxin B hemoperfusion in patients with septic shock: a propensity-matched cohort study. Critical care (London, England).

[59] Kodama Y, Takahashi G, Kan S, Masuda T, Ishibe Y, Akimaru R (2019). Use of Direct Hemoperfusion with Polymyxin B-Immobilized Fiber for the Treatment of Septic Shock Complicated with Lemierre Syndrome Caused by Fusobacterium necrophorum. Case reports in critical care.

[60] (2020). US FDA Approves an Investigational Device Exemption for Spectral Medical PMX to Treat COVID-19 Patients Suffering from Septic Shock.

